# Effects of Chronic Elevation in Plasma Membrane Cholesterol on the Function of Human Na^+^/Taurocholate Cotransporting Polypeptide (NTCP) and Organic Cation Transporter 1 (OCT1)

**DOI:** 10.3390/livers5030045

**Published:** 2025-09-12

**Authors:** Jessica Y. Idowu, Caylie McKimens, Bruno Hagenbuch

**Affiliations:** Department of Pharmacology, Toxicology, and Therapeutics, The University of Kansas Medical Center, Kansas City, KS 66160, USA

**Keywords:** NTCP, OCT1, transporter, cholesterol, fatty liver

## Abstract

**Background:**

We have previously demonstrated that the function and expression of the Na^+^/taurocholate cotransporting polypeptide (NTCP) and the organic cation transporter 1 (OCT1) are affected by increasing free or unesterified cholesterol in the plasma membrane by an acute incubation with cholesterol for 30 min. In the current study we wanted to extend these findings to a more chronic condition to mimic what would be seen in obese patients.

**Methods:**

We incubated HEK293 cells that stably express NTCP or OCT1 for 24 h with 0.05 mM cholesterol and determined their function by measuring uptake of radio-active taurocholate or MPP^+^. Expression at the plasma membrane was quantified with a biotinylation assay combined with Western blots.

**Results:**

Incubation with cholesterol increased the cholesterol content of the cells by about 2-fold. Transport mediated by NTCP and OCT1 was decreased. Membrane expression for both transporters showed a slight decrease, and when kinetics were normalized for the membrane expression, the V_max_ for NTCP-mediated taurocholate uptake slightly decreased, but the V_max_ and the capacity (V_max_/K_m_) for OCT1-mediated MPP^+^ uptake increased by 2.5-fold and 3-fold, respectively. Acyl-Coenzyme A acyltransferase inhibitors enhanced the decrease in transport function, potentially due to retention of more free cholesterol in the plasma membrane.

**Conclusions:**

Chronic increases in free cholesterol in the plasma membrane can result in increased or decreased transporter function and expression. In the case of OCT1, which is involved in the uptake of the anti-diabetic drug metformin into hepatocytes, the 3-fold increase in transport capacity might affect drug therapy.

## 1. Introduction

Increases in free cholesterol content are a hallmark of obesity and are associated with metabolic dysfunction-associated steatotic liver disease (MASLD) and metabolic dysfunction-associated steatohepatitis (MASH) [1,2]. By free cholesterol we mean cholesterol that is not attached to a fatty acid, and thus, unesterified. The onset of MASLD and MASH, along with the increase in cholesterol content, is a gradual process and has been linked to various disease states, including hypercholesterolemia, cardiovascular and neurodegenerative diseases, and cancer [3-7]. The Na^+^/taurocholate cotransporting polypeptide (NTCP; *SLC10A1*) and the organic cation transporter 1 (OCT1; *SLC22A1*) are expressed at the basolateral membrane of human hepatocytes [8,9]. NTCP maintains the enterohepatic circulation of bile acids [10-12], but can also transport conjugated steroid hormones [13,14] and drugs like pitavastatin, rosuvastatin, and fluvastatin [15-18]. OCT1 is an organic cation transporter that mediates the uptake of numerous endo- and xenobiotic organic cations, including the drug metformin, which is frequently taken by patients with MASLD, particularly those with type 2 diabetes [19,20]. Thus, changes in the function of these transporters due to increased levels of free cholesterol in the plasma membrane could affect drug therapy and, thus, treatment outcome. We have previously demonstrated that an acute 30 min increase in cholesterol content impaired NTCP- and OCT1-mediated substrate uptake [21]. In the present study, we expanded our analysis to a more chronic situation by investigating the effect of increased concentrations of free cholesterol for 24 h, more reminiscent of humans with fatty liver disease, on NTCP and OCT1 function and localization.

## 2. Materials and Methods

### 2.1. Experimental Materials

Radiolabeled [^3^H]-Taurocholic acid was purchased from Revvity (Waltham, MA, USA), and radiolabeled [^3^H]-Methyl-4-phenylpyridinium iodide (MPP^+^) from American Radiolabeled Chemicals, Inc. (St. Louis, MO, USA). Water-soluble cholesterol (cholesterol balanced with methyl-β-cyclodextrin) and the Acyl-Coenzyme A acyltransferase (ACAT) inhibitors Sandoz 58-035, Avasimibe, and Cl976 were obtained from Sigma Aldrich (St. Louis, MO, USA).

Mouse anti-Alpha 1 Sodium Potassium ATPase antibody (Ab7671, 1:2000) was from Abcam (Waltham, MA, USA), and rabbit anti-Flotillin-1 antibody (F1180, 1:2000) was obtained from Sigma Aldrich. Mouse anti-Clathrin Heavy Chain antibody (610499, 1:1000) was purchased from BD Biosciences (Franklin Lakes, NJ, USA). Mouse Tetra His antibody (34670, 1:2000) was bought from QIAGEN (Germantown, MD, USA). The mouse anti-SLC22A1 antibody (NBP1-51684, 1:1000) was obtained from Novus Biologicals (Littleton, CO, USA). HRP-conjugated goat anti-mouse (31430, 1:10,000) or anti-rabbit (A16096, 1:10,000) secondary antibodies were from Thermo Fisher Scientific (Carlsbad, CA, USA).

### 2.2. Cell Culture, Cholesterol Treatment, and Uptake Experiments

HEK293 cells stably expressing His-tagged NTCP and OCT1 and empty vector-transfected control cells were cultured and maintained as described previously [22,23].

To mimic chronic cholesterol conditions, the cells were incubated in DMEM containing lipid-depleted fetal bovine serum (Biowest USA, Bradenton, FL, USA, #S148L) supplemented with 0.05 mM cholesterol (cholesterol water-soluble, Sigma Aldrich) for 24 h. For the treatment with ACAT inhibitors, either 12.5 μg/mL Sandoz 58-035, 10 μM Avasimibe, or 10 μM Cl-976 was added. To measure uptake, the HEK293 cells were plated on poly-D-lysine-coated plates or in the presence of 2 μg/μL poly-ethylenimine [24]. NTCP-mediated uptake of [^3^H]-taurocholate and OCT1-mediated uptake of [^3^H]-MPP^+^ were measured as described previously [21].

For kinetic determinations, uptake was performed at 15 s under initial linear rate conditions for NTCP and OCT1 at 37 °C.

### 2.3. Cholesterol Quantification

The Amplex^™^ Red Cholesterol Assay Kit from Thermo Fisher Scientific was used following the manufacturer’s instructions. Fluorescence was measured at an excitation of 540 nm and an emission of 620 nm.

### 2.4. Surface Biotinylation and Western Blotting

The HEK293 cells were grown at 800,000 cells/well on 6-well plates, and after the respective treatments, biotinylated proteins were isolated and analyzed as previously described [21,25].

### 2.5. Statistical Analysis

Data were analyzed using one-way ANOVA followed by “Dunnett’s multiple comparisons test” in GraphPad Prism 10 (GraphPad Software Inc., San Diego, CA, USA). Significance and *p*-values are given in the figure legends.

## 3. Results

### 3.1. Functional Consequences of a Chronic Increase in Plasma Membrane Free Cholesterol Levels

**Figure 1.**
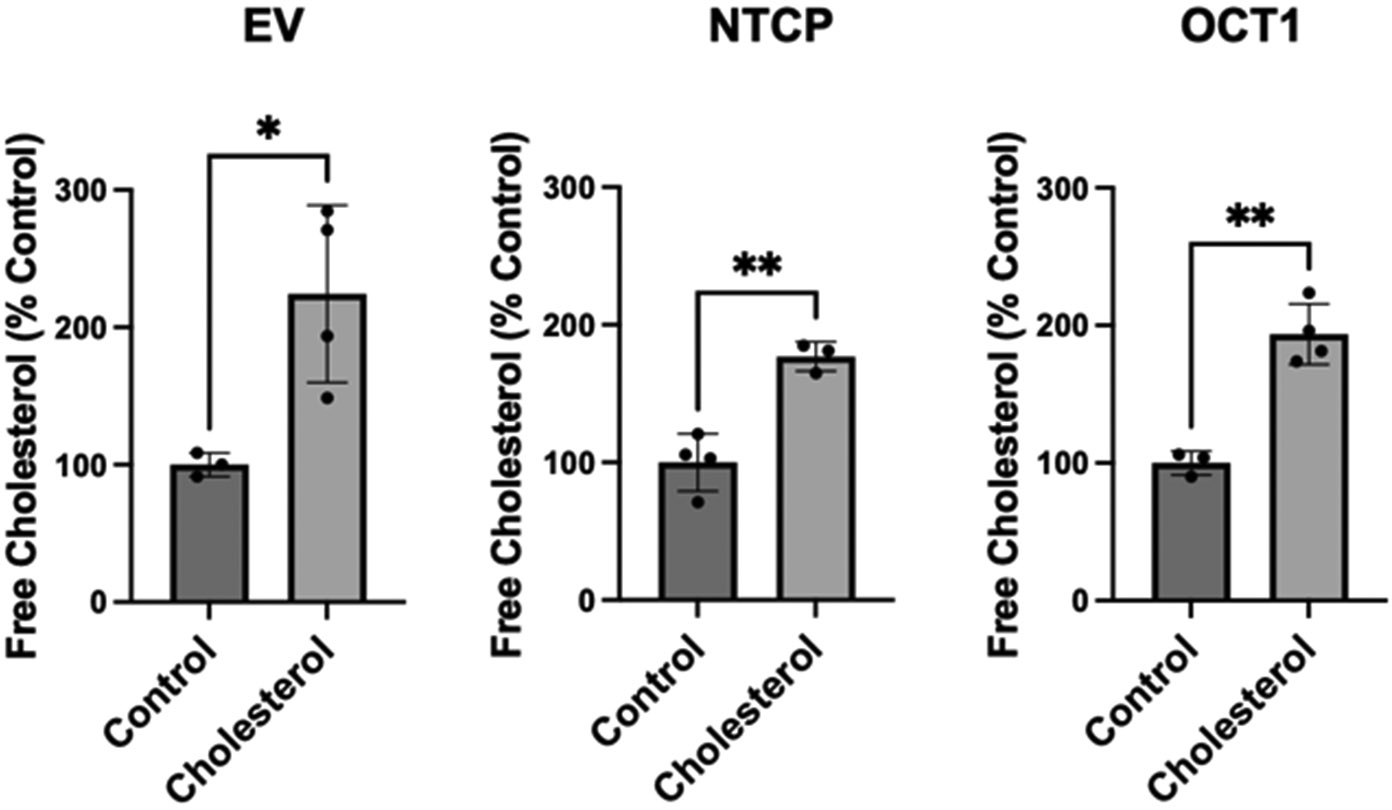
Free cholesterol content in stably expressing HEK293 cells. The empty vector (EV)-transfected and NTCP- and OCT1-expressing HEK293 cells were treated for 24 h in media containing delipidated FBS (control) or 0.05 mM cholesterol. The graphs represent the mean ± SD of at least 3 independent experiments; * *p* < 0.05 and ** *p* < 0.01 compared to control.

To assess the impact of chronic cholesterol exposure on transporters, we established a cholesterol concentration and exposure time that did not impact cell viability or cell adhesion for functional studies. Initial experiments demonstrated that after a 24 h treatment with 0.05 mM cholesterol, the NTCP- and OCT1-expressing HEK293 cells still adhered to the plates, while cells lifted off the plates at longer time points or higher cholesterol concentrations. We measured free cholesterol levels of the NTCP- and OCT1-expressing HEK293 cells to quantify the increase in plasma membrane-free cholesterol. The impact of a 24 h exposure to 0.05 mM cholesterol was determined using the Amplex Red Cholesterol Assay Kit without cholesterol esterase. Figure 1 demonstrates that in the empty vector-transfected HEK293 cells, free cholesterol increased by 124%; in the NTCP-expressing cells, cholesterol increased by 75%; and in the OCT1-expressing cells, the increase was 94%. Thus, we concluded that a 24 h incubation with 0.05 mM cholesterol can almost double the free cholesterol levels in NTCP- and OCT1-expressing HEK293 cells. This increase in free cholesterol content of the transporter-expressing cells after a 24 h 0.05 mM cholesterol treatment was similar to what we determined previously for an acute condition where cells were incubated for 30 min with much higher concentrations of free cholesterol (0.8 mM for NTCP and 0.4 mM for OCT1) [21].

**Figure 2.**
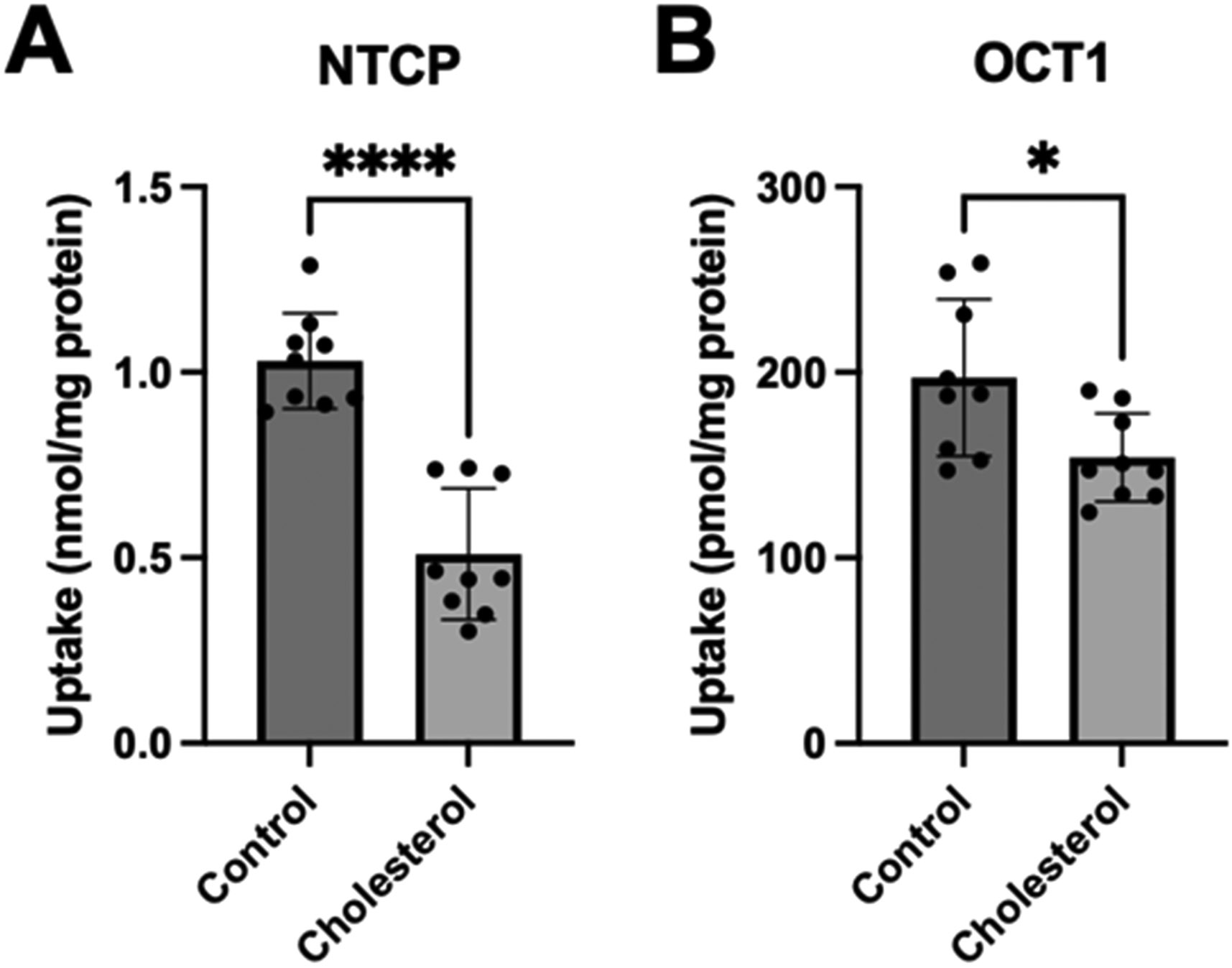
Transporter-mediated substrate uptake following chronic cholesterol exposure. Uptake of (**A**) (100 μM) [^3^H]-taurocholate for the NTCP- and (**B**) (16.7 nM) [^3^H]-MPP^+^ for the OCT1-expressing HEK293 cells was measured for 30 s at 37 °C, 24 h after treatment with 0.05 mM cholesterol. Net uptake was determined by subtracting the uptake measured in a sodium-free buffer from the uptake measured in a sodium-containing buffer for NTCP, or by subtracting the uptake of the empty vector (EV)-transfected cells from the OCT1-expressing cells for OCT1. The results are reported as the mean ± SD of three independent experiments performed with triplicate determinations: * *p* < 0.05 and **** *p* < 0.0001.

To determine what impact the chronic increase in free cholesterol would have on the function of NTCP and OCT1, we measured substrate uptake. After a 24 h incubation in the presence of 0.05 mM cholesterol, uptake of 100 μM taurocholate for the NTCP-expressing cells and 16.7 nM methyl-4-phenylpyridinium iodide (MPP^+^) for the OCT1-expressing cells was measured. The results shown in Figure 2 demonstrate that increasing the cholesterol content decreased uptake mediated by both transporters compared to the control. These effects were consistent with those observed following the acute cholesterol incubation reported previously [21]; however, the decrease in the OCT1-mediated uptake was not as pronounced.

### 3.2. Characterizing the Effect of a Chronic Increase in Free Cholesterol on Transporter Expression and Function

**Figure 3.**
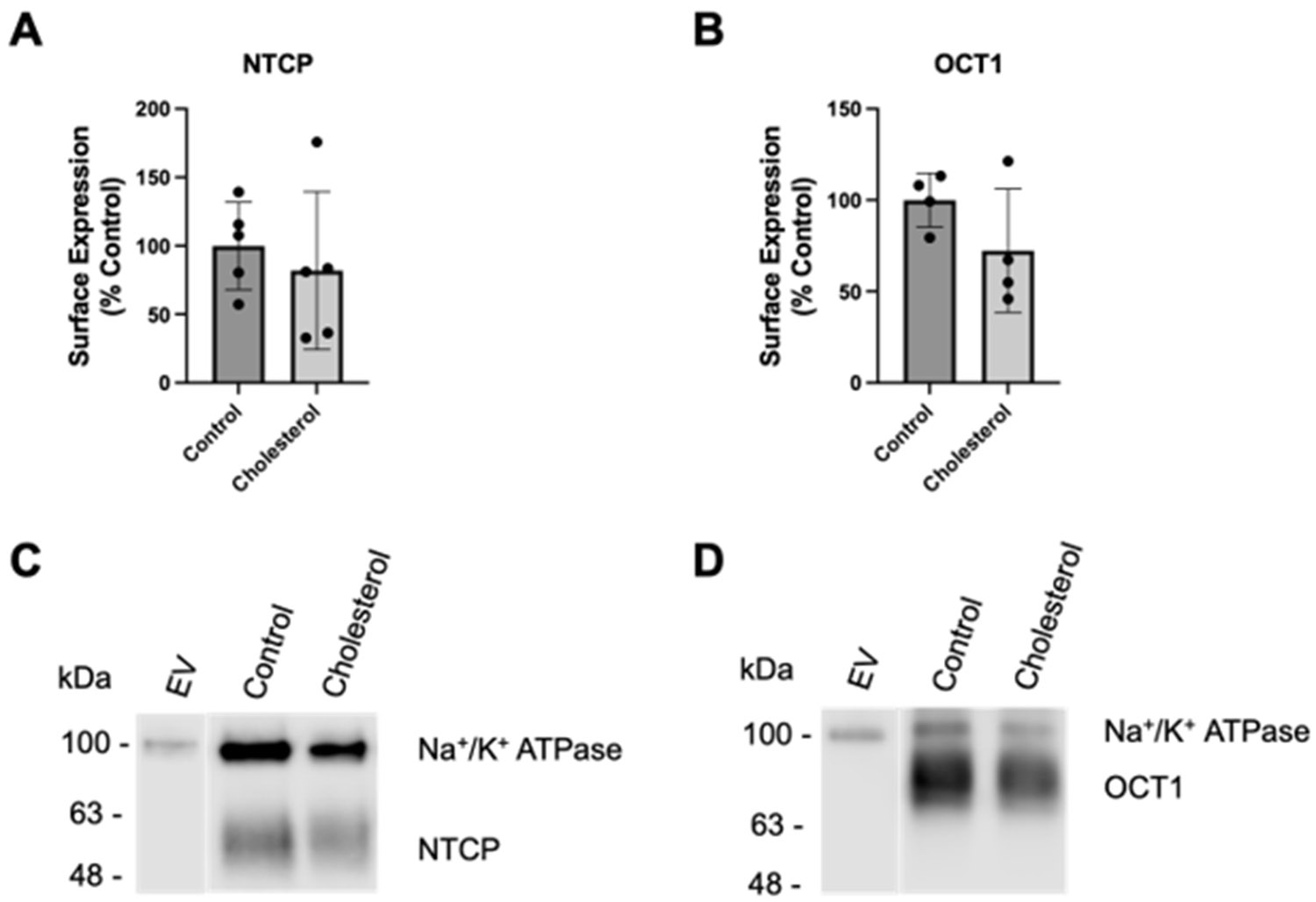
Surface expression of transporters following chronic cholesterol exposure. Plasma membrane surface expression was quantified for the (**A**) NTCP- and (**B**) OCT1-expressing HEK293 cells. The cells were treated for 24 h with DMEM containing lipid-depleted FBS (control), or 0.05 mM cholesterol. Surface expression for (**C**) NTCP and (**D**) OCT1 is depicted using a representative Western blot. The results are displayed as percent of control and reported as the mean ± SD of five (NTCP) or four (OCT1) independent experiments.

We previously reported that increasing the plasma membrane free cholesterol content acutely during a 30 min incubation with 0.8 mM or 0.4 mM cholesterol for NTCP and OCT1, respectively, resulted in decreased transporter surface expression [21]. To investigate how chronic elevation of cholesterol would affect the plasma membrane expression of NTCP and OCT1, we assessed transporter expression at the plasma membrane after a 24 h incubation with 0.05 mM cholesterol using surface biotinylation and Western blot. Expression of both transporters tended towards a decrease (Figure 3). NTCP expression was about 20% lower after cholesterol treatment (Figure 3A), while OCT1 was about 30% lower (Figure 3B). Representative Western blots for NTCP and OCT1 surface expression are shown in Figure 3C,D, with Na^+^/K^+^ ATPase as the loading control. The diminished surface expression of NTCP and OCT1 was used to normalize the transporter function in the following experiments.

**Figure 4.**
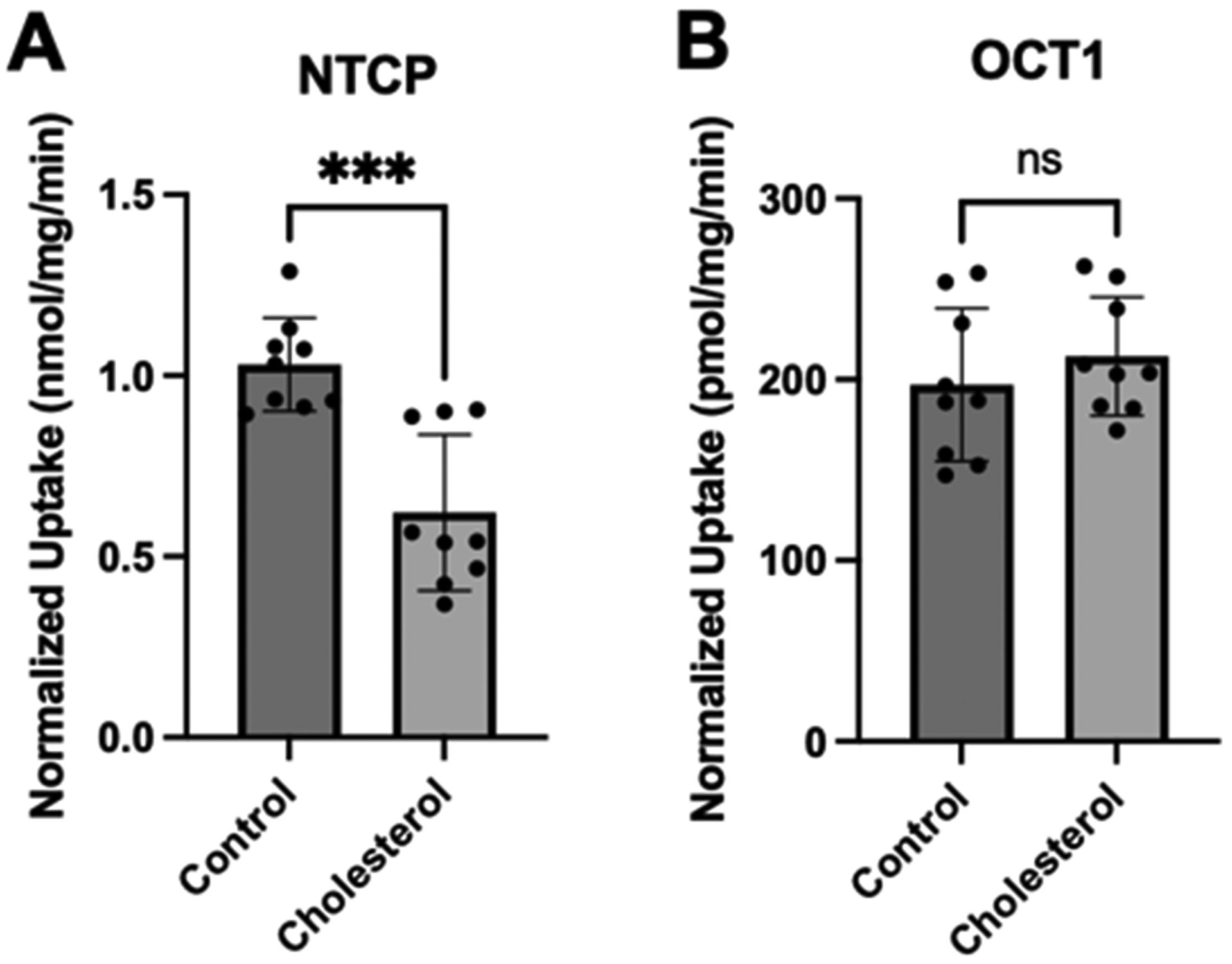
Normalized transporter-mediated substrate uptake following chronic cholesterol exposure. Uptake of (**A**) (100 μM) [^3^H]-taurocholate for the NTCP- and (**B**) (16.7 nM) [^3^H]-MPP^+^ for the OCT1-expressing HEK293 cells 24 h after treatment with 0.05 mM cholesterol. Transport shown in Figure 2 was corrected for surface expression. The graphs represent the mean ± SD of 3 independent experiments performed with triplicate determinations: *** *p* < 0.001.

After normalization, the NTCP-mediated taurocholate uptake was still significantly lower than the control (Figure 4A). However, the normalized OCT1-mediated MPP^+^ uptake was similar to the control values (Figure 4B). These findings are very similar to what we saw following a 30 min incubation at the higher cholesterol concentrations [21].

**Figure 5.**
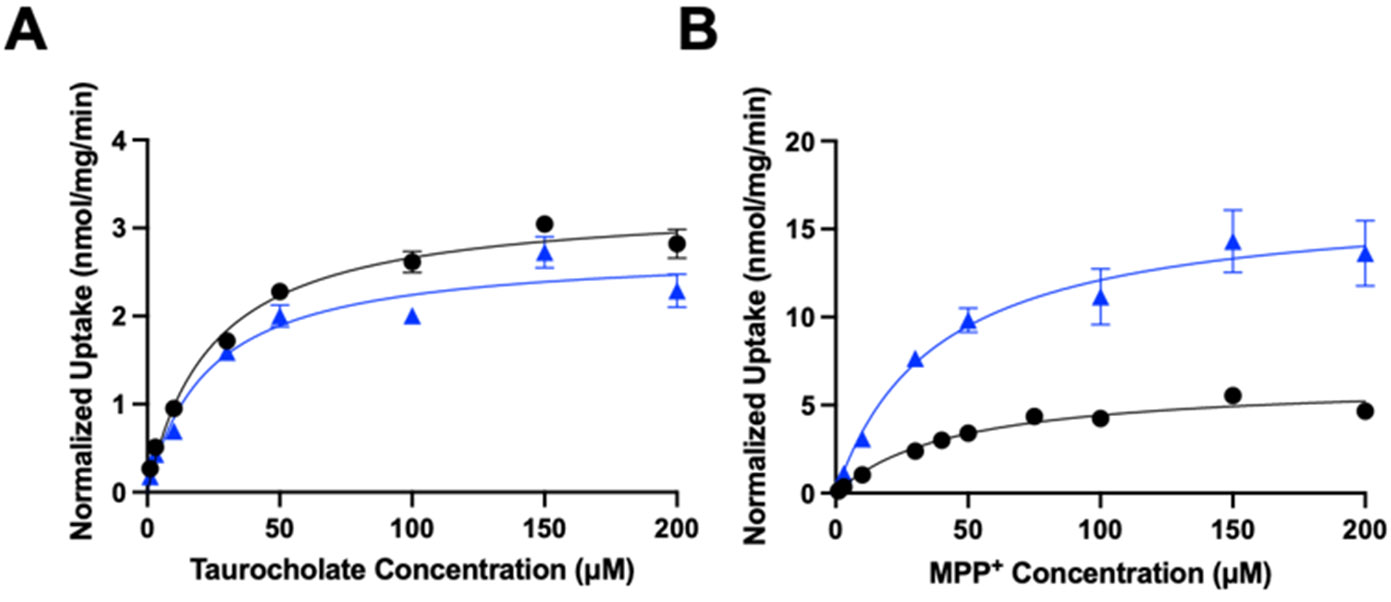
Normalized kinetics of transporter-mediated substrate uptake following chronic cholesterol exposure. Uptake of increasing concentrations of (**A**) taurocholate or (**B**) MPP^+^ was measured at 37 °C for 15 s in the NTCP-expressing or OCT1-expressing HEK293 cells, respectively, and corrected for protein expression. Net uptake was calculated as mentioned previously. Values are means ± SEM of three independent experiments performed in triplicate. Circles represent the control conditions and triangles the added cholesterol conditions. The Michaelis–Menten equation in GraphPad Prism was used to fit the curves.

To further characterize the functional impact of chronic cholesterol exposure on NTCP- and OCT1-mediated uptake, we performed kinetic experiments. Time-dependent experiments revealed that uptake for NTCP and OCT1 was linear at least for 15 s. Therefore, concentration-dependent uptake was measured after 24 h of 0.05 mM cholesterol treatment at 15 s. Figure 5 and Table 1 summarize the results.

The control NTCP-mediated taurocholate uptake was saturable with a K_m_ of 24 ± 4.0 μM and a V_max_ of 3.3 ± 0.1 nmol/mg/min. Following treatment with 0.05 mM cholesterol, the K_m_ stayed the same at 22.7 ± 6.7 μM while the V_max_ decreased slightly to 2.7 ± 0.2 nmol/mg/min (Table 1). For the OCT1-mediated uptake of MPP^+^, the control uptake saturated with a K_m_ value of 44.4 ± 9.7 μM and a V_max_ of 6.4 ± 0.5 nmol/mg/min. Treatment with 0.05 mM cholesterol did not affect the K_m_ value (38.0 ± 6.3 μM) but increased the V_max_ to 16.7 ± 0.9 nmol/mg/min (Table 1). Thus, for NTCP, there were no significant changes in the kinetic parameters, while for OCT1, the V_max_ increased by about 2.5-fold, resulting in an almost 3-fold higher capacity (Table 1).

### 3.3. Effect of ACAT Inhibitors on Function and Expression of NTCP and OCT1

**Figure 6.**
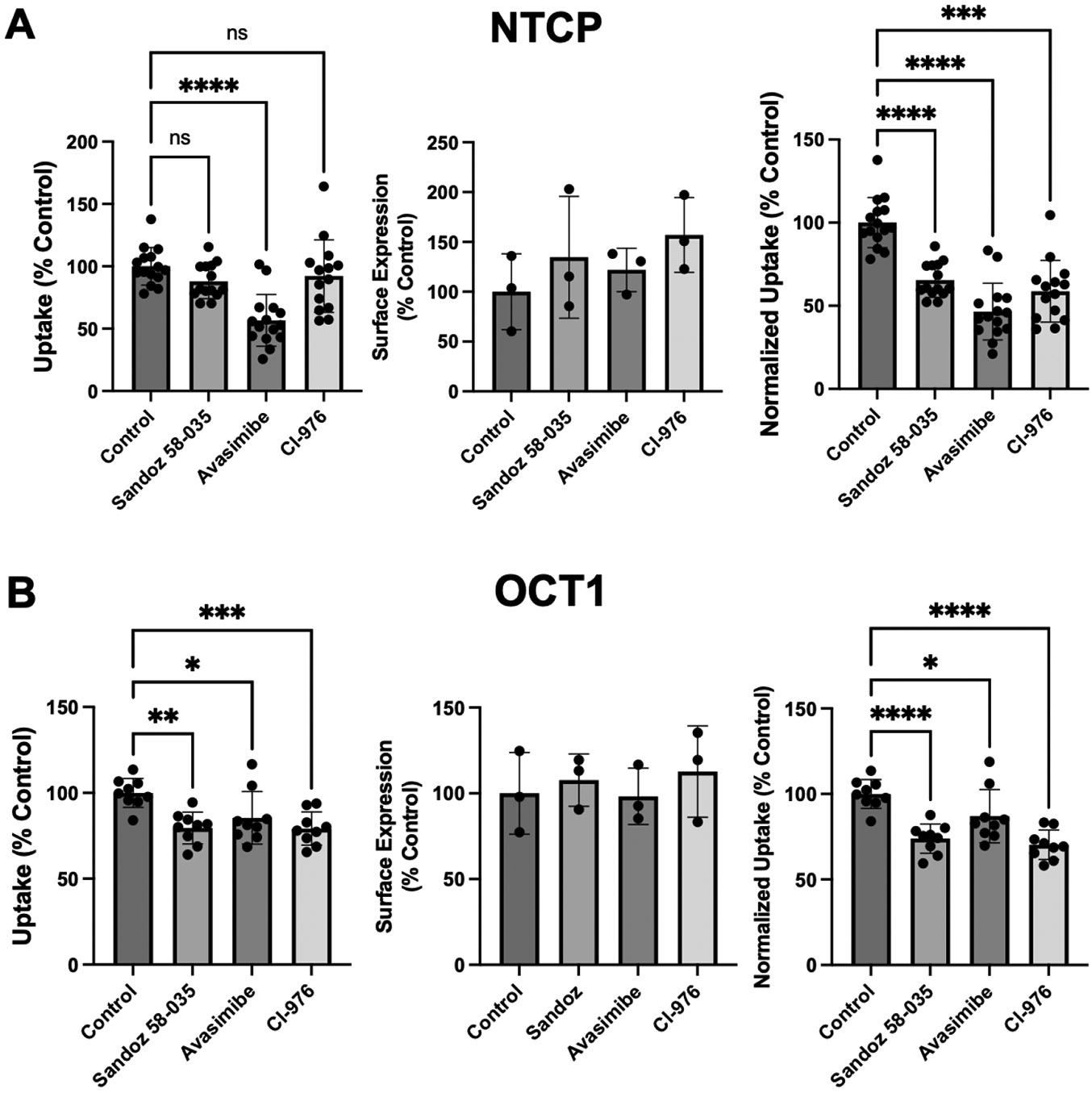
Effect of ACAT inhibitors on the function and expression of (**A**) NTCP and (**B**) OCT1 after treatment with 0.05 mM for 24 h. The cells were treated for 24 h with 0.05 mM cholesterol in DMEM containing lipid-depleted FBS, in the absence (control) or presence of the three ACAT inhibitors Sandoz 58-035 (12.5 μg/mL), Avasimibe (10 μM), or Cl-976 (10 μM). After the treatment, uptake of 100 μM [^3^H]-taurocholate (NTCP) or 16.7 nM [^3^H]-MPP^+^ (OCT1) was measured (left panels). Using surface biotinylation, the transporter expression at the plasma membrane was quantified (middle panel) and used to normalize uptake (right panel). Three to five independent experiments were performed. Mean ± SD is shown. * *p* < 0.05, ** *p* < 0.01, *** *p* < 0.001, **** *p* < 0.0001.

Incubation with 0.05 mM cholesterol for 24 h could result in the formation of cholesterol esters and reduce the concentration of free cholesterol in the plasma membrane. To prevent such esterification, Acyl-Coenzyme A acyltransferase (ACAT) inhibitors were included during the 24 h incubation with cholesterol. We used three different ACAT inhibitors: Sandoz 58-035 (12.5 μg/mL) [26], Avasimibe (10 μM) [27], or Cl-976 (10 μM) [28]. As shown in Figure 6, treatment with all three ACAT inhibitors resulted in decreased substrate uptake by both transporters.

## 4. Discussion

We previously demonstrated that an acute increase (30 min) in free cholesterol slightly increased substrate affinity and decreased V_max_ for NTCP, resulting in a decreased transport capacity. For OCT1, the effect on the K_m_ value was more pronounced, and overall, the capacity clearly decreased [21]. In the present study we wanted to expand these findings to a more chronic situation where we incubated the cells with free cholesterol for 24 h, more closely mimicking the situation of patients with MASLD or MASH [2].

The major findings of these more chronic conditions are the following: increasing the free cholesterol concentration for 24 h resulted in a trend towards decreased K_m_ and V_max_, and consequently towards a decrease in the transport capacity of NTCP, similar to what we have reported for the acute condition [21]. In contrast, for OCT1, increasing cholesterol for 24 h resulted in a 3-fold increase in capacity, which was opposite to the acute condition. This result was mainly driven by an increase in V_max_, which reflects an increased intrinsic transport activity per surface-expressed transporter since the results were normalized for protein at the plasma membrane. Patients with metabolic syndrome or MAFLD are frequently treated with metformin for their type 2 diabetes. Therefore, an increased transport capacity of OCT1 suggests that metformin would be taken up into hepatocytes more efficiently in patients with increased free cholesterol, and therapeutic doses in such patients might have to be adjusted.

Our findings with NTCP and OCT1, where increasing cholesterol in the cell membrane had different effects, are consistent with previous published studies. Bastiaanse et al. (1997), who reviewed the effects of membrane cholesterol on transport processes in the plasma membrane [29], found that increasing the cholesterol in the plasma membrane decreased the function of ATPases but increased the function of most other transporters studied. More recently, Scanga et al. (2025) reported that an increase in cholesterol resulted in an increase in the V_max_ for histidine transport by purified LAT1 in proteoliposomes [30]. Given that increasing free cholesterol in the membrane results in lower fluidity or higher rigidity [31], the increase in the turnover for OCT1 is kind of unexpected. One would expect that a transmembrane protein would turn over quickly at a lower rigidity or higher fluidity. However, it has to be taken into consideration that the observed changes could be due to different localization of the transporters in lipid rafts or plasma membrane microdomains, as we saw after a 30 min incubation with cholesterol [21], and that the lipid composition of the plasma membrane changes with the increased cholesterol content.

Another possibility is that cholesterol interacts directly with the transport proteins, and we will investigate this possibility in future experiments. Previous studies identified a cholesterol recognition/interaction amino acid consensus sequence (CRAC) as (L/V)-X1-5-(F/Y/W)- X1-5-(K/R) [32,33] or its reverse as CARC. Recently, such a CRAC was identified in OCT2, an organic cation transporter mainly expressed in the kidney [34]. Preventing cholesterol interaction by site-directed mutagenesis abolished allosteric features of OCT2 due to cholesterol binding. This identified CRAC sequence is highly conserved in OCT1. Therefore, in future experiments, we will investigate whether the effect of cholesterol on OCT1 function is the same after the CRAC sequence has been changed using site-directed mutagenesis.

Loading the plasma membrane with free cholesterol can result in esterification of cholesterol and storage of the esterified cholesterol in lipid droplets [35]. To prevent this esterification and investigate the effect of its inhibition, we added three different ACAT inhibitors during the 24 h incubation with cholesterol. For both transporters, a further decrease in normalized function was seen. One possible interpretation of these findings is that due to the inhibition of ACAT, more free cholesterol stays in the plasma membrane, and the effects on transporter function are enhanced. Another option is that these ACAT inhibitors have a direct effect on the transporter and act as acute inhibitors. In preliminary experiments, we did not see any inhibition of OCT1-mediated uptake but a clear inhibition of NTCP-mediated taurocholate uptake (Figure S1). Future experiments will be needed to determine the type and potency of inhibition. It is interesting to note that Avasimibe was evaluated as a lipid-lowering drug back in the 1990s, but clinical trials did not result in positive outcomes [36]. However, Post et al. (1999) reported that in rats, Avasimibe stimulated bile acid synthesis and 7alpha-hydroxylase activity [37]. This stimulation of bile acid synthesis could potentially, at least in part, be explained by the inhibition of NTCP-mediated bile acid uptake, leading to lower intracellular bile acid concentrations and thus stimulating the conversion of cholesterol to bile acids.

The presented studies have the limitation that the experiments were performed in HEK293 cells that expressed NTCP or OCT1 and not in human hepatocytes. While HEK293 cells are a great tool to express individual transporters and manipulate growth conditions, they have several disadvantages compared to human hepatocytes. First, they only express a limited number of hepatocellular transporters, normally only one transporter at a time, and frequently this transporter is overexpressed compared to hepatocytes. This prevents potential effects from the interactions of multiple transporters that are expressed in the same hepatocyte. Second, HEK293 cells do not express all the metabolizing enzymes that human hepatocytes express. Consequently, especially under the more chronic conditions used in this study, potential effects of such liver enzymes are missing in HEK293 cells. And third, compared to the situation in the liver, where hepatocytes interact with Kupffer cells, stellate cells, and potentially other cell types, HEK293 cells in culture cannot replicate these more complex interactions. Unfortunately, we could not plate human hepatocytes that were isolated from fatty livers, because they did not adhere to the plates. We are in the process of developing HepG2 cells that express these transporters via activation of the intrinsic promoter. Such HepG2 cells would be more hepatocyte-like cells than the HEK293 cells, and the results should better represent what would be expected from human hepatocytes.

In conclusion, increasing the free cholesterol content in the plasma membrane for 24 h, mimicking a more chronic situation than the previously reported 30 min incubation, resulted in a 3-fold increase in the capacity of OCT1 to transport its substrate. These findings suggest that drug therapy in patients treated with OCT1-transported drugs might be affected. For NTCP, the effects were not as pronounced as during the 30 min acute treatment. Interestingly, treating the cells with Avasimibe, which was a lipid-lowering drug candidate, resulted in a significant reduction in NTCP-mediated taurocholate transport. Future experiments in a more hepatocyte-like cell line or in MASLD animal models will be needed to explain the underlying mechanisms and further elucidate potential consequences for patients with obesity and type 2 diabetes.

## Supplementary Material

Suppl. Figures

**Supplementary Materials:** The following supporting information can be downloaded at https://www.mdpi.com/article/doi/s1. Figure S1: Acute effect of ACAT inhibitors on the uptake mediated by NTCP and OCT1.

## Figures and Tables

**Table 1.** Kinetic parameters for the NTCP- and OCT1-expressing HEK293 cells. Kinetic parameters, K_m_, V_max_, and the capacity (V_max_/K_m_), were calculated using the Michaelis–Menten equation in GraphPad Prism and are reported as the mean ± SEM of at least three independent experiments performed in triplicate; * *p* < 0.05 as compared to the control condition.

Transporter	Parameters	Control	Cholesterol
NTCP	K_m_ (μM)	24.0 ± 4.0	22.7 ± 6.7
	V_max_ (nmol/mg/min)	3.3 ± 0.1	2.7 ± 0.2
	V_max_/K_m_ (μL/mg/min)	138 ± 23	119 ± 36
OCT1	K_m_ (μM)	44.4 ± 9.7	38.0 ± 6.3
	V_max_ (nmol/mg/min)	6.4 ± 0.5	16.7 ± 0.9
	V_max_/K_m_ (μL/mg/min)	144 ± 33	439 ± 77 *

## Data Availability

All data are contained within the article and Supplementary Materials.
