# Supplementary material for: Effects of Chronic Elevation in Plasma Membrane Cholesterol on the Function of Human Na+/Taurocholate Cotransporting Polypeptide (NTCP) and Organic Cation Transporter 1 (OCT1)
Source: Livers. Author manuscript; Available in PMC 2025 Dec 10. (PMC12687901; doi:10.3390/livers5030045)

Supplementary information to:

Effects of Chronic Elevation in Plasma Membrane Cholesterol on the Function of Human Na$^+$/Taurocholate Cotransporting Polypeptide (NTCP) and Organic Cation Transporter 1 (OCT1)

Jessica Y. Idowu, Caylie McKimens and Bruno Hagenbuch

Department of Pharmacology, Toxicology, and Therapeutics, The University of Kansas Medical Center, Kansas City, KS 66160, USA

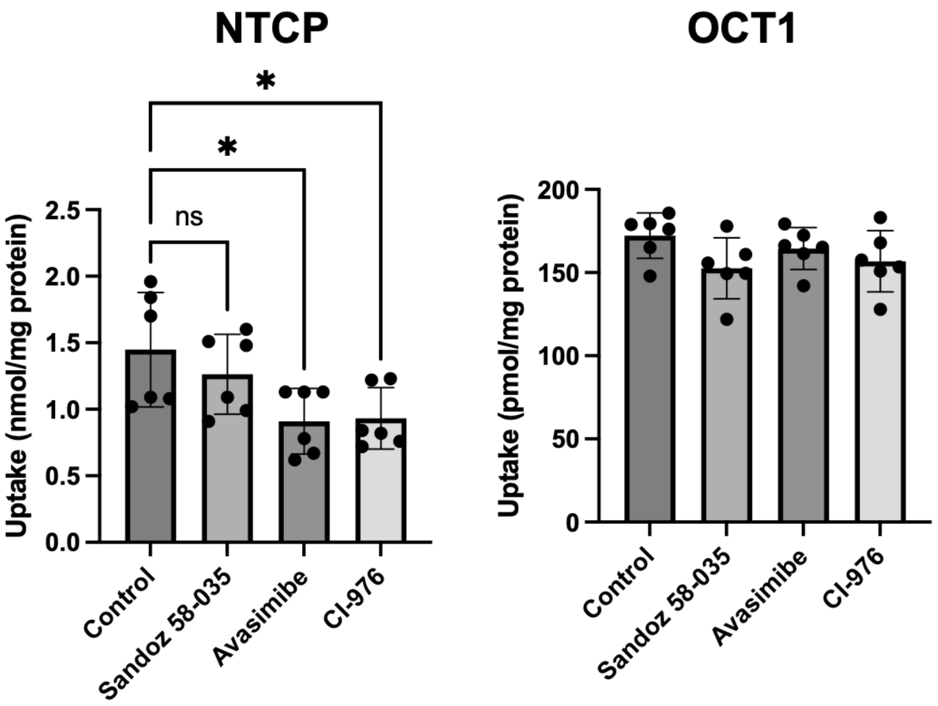

Figure S1. Acute effect of ACAT inhibitors on the uptake mediated by NTCP and OCT1. Uptake into cells expressing NTCP or OCT1 was measured for 30 seconds with 100 µM [^{3}H]-taurocholate (NTCP) or 16.7 nM [^{3}H]-MPP$^+$ (OCT1) in the absence (control) or presence of the indicated ACAT inhibitors: Sandoz 58-035 (12.5 µg/mL), Avasimibe (10 µM), or Cl-976 (10 µM). Net uptake was determined by subtracting the uptake measured in a sodium-free buffer from the uptake measured in a sodium-containing buffer for NTCP, or by subtracting the uptake of empty vector transfected cells from OCT1-expressing cells for OCT1. Results are reported as the mean ± SD of two (for NTCP) and three (for OCT1) independent experiments performed with triplicate determinations: * $p < 0.05$.

Western blots for the quantification of NTCP in Figure 3

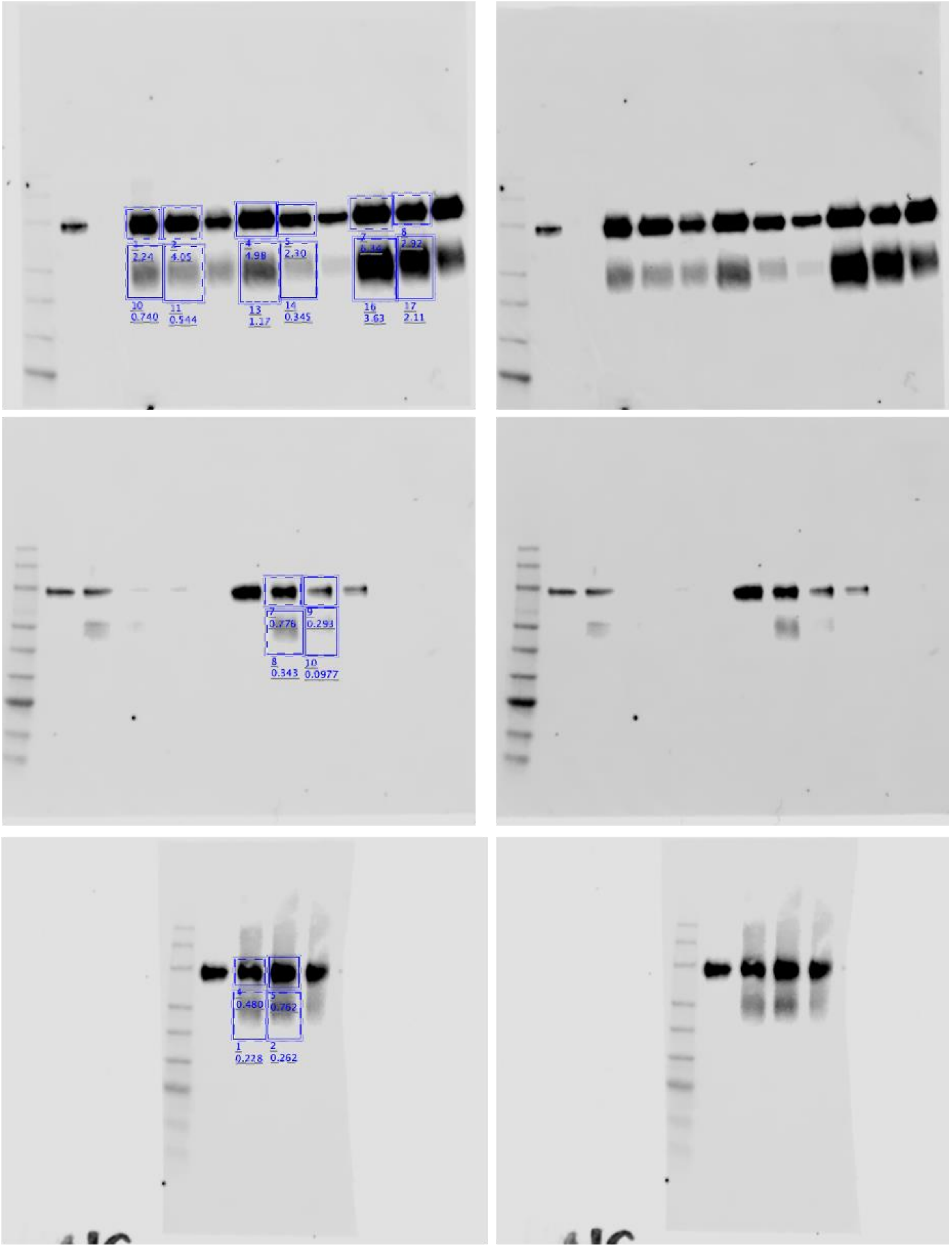

Western blots for the quantification of OCT1 in Figure 3

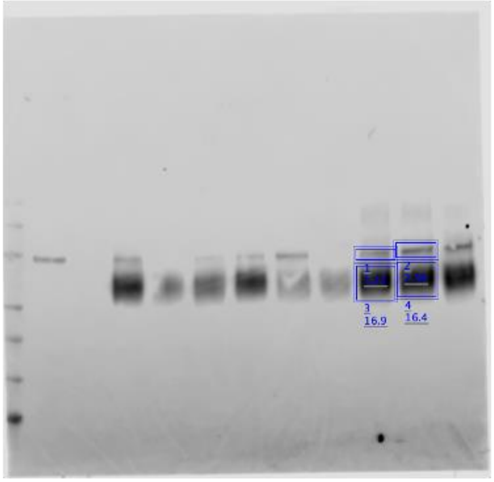

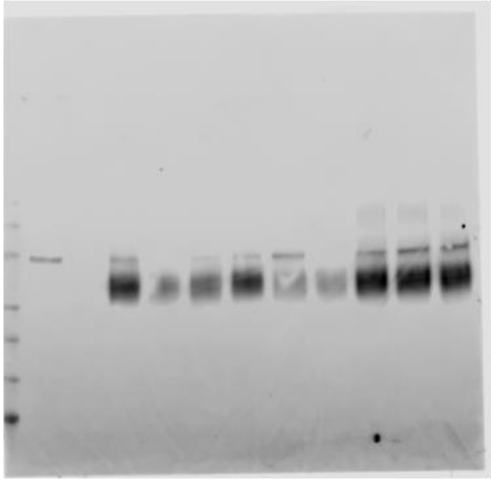

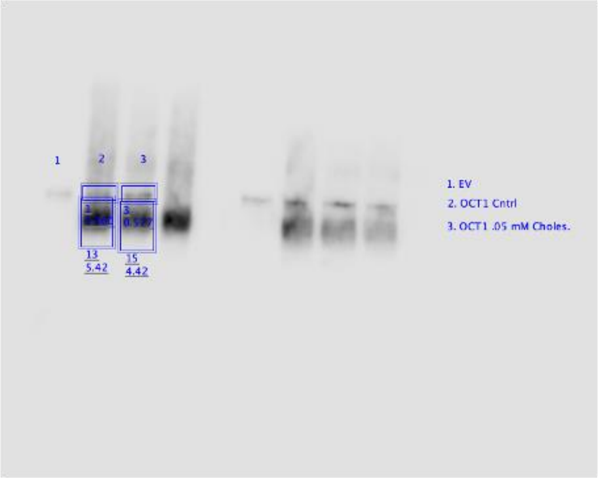

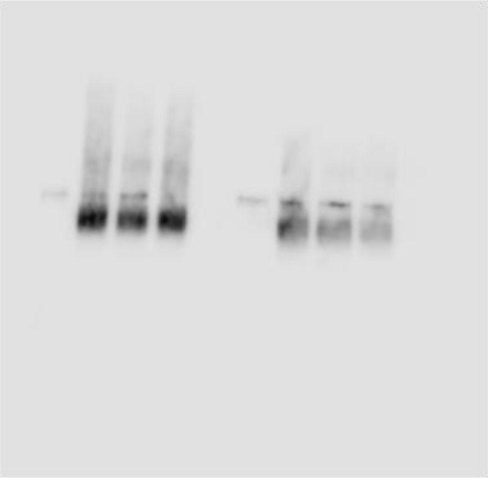

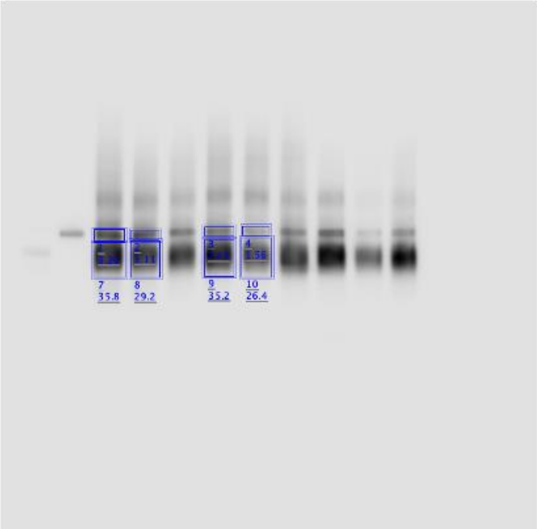

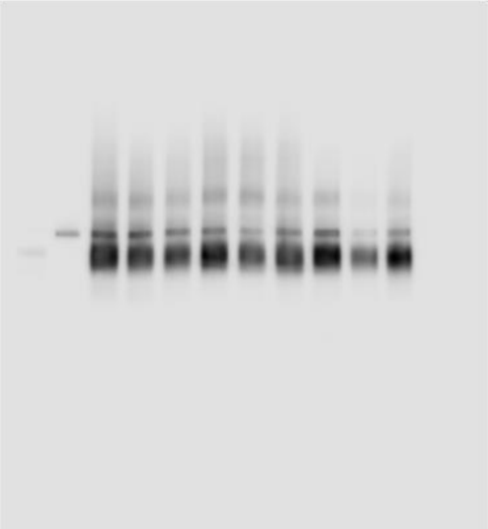

Supplement: Suppl. Figures [file NIHMS2124959-supplement-Suppl__Figures.pdf]
